# Impact of COVID-19 Pandemic on Food Insecurity in an Urban Emergency Department Patient Population

**DOI:** 10.5811/westjem.2023.1.59007

**Published:** 2023-02-26

**Authors:** Donya Enayati, Virginia Chan, Gavin Koenig, Kathryn Povey, Heng Ky Nhoung, Les R. Becker, Kacie J. Saulters, Rebecca Breed, Yumi Jarris, Thomas Zarembka, Michelle Magee, Munish Goyal

**Affiliations:** * Georgetown University School of Medicine, Washington, DC; † MedStar Washington Hospital Center, Department of Emergency Medicine, Washington, DC; ‡ MedStar Georgetown University Hospital, Department of Internal Medicine, Washington, DC; § MedStar Georgetown University Hospital, Department of Family Medicine, Washington, DC; ¶ Food and Friends Program, Washington, DC; || MedStar SiTEL, Washington, DC; # University of Maryland Capital Region Health, Department of Internal Medicine, Largo, Maryland; ** MedStar Health, MedStar Diabetes Institute, Washington, DC

## Abstract

**Introduction:**

Food insecurity (FI) has been associated with adverse health outcomes and increased healthcare expenditures. Many families experienced reduced access to food during the coronavirus disease 2019 (COVID-19) pandemic. A 2019 study revealed that the pre-pandemic prevalence of FI at an urban, tertiary care hospital’s emergency department (ED) was 35.3%. We sought to evaluate whether the prevalence of FI in the same ED patient population increased during the COVID-19 pandemic.

**Methods:**

We performed a single-center, observational, survey-based study. Surveys assessing for FI were administered to clinically stable patients presenting to the ED over 25 consecutive weekdays from November–December 2020.

**Results:**

Of 777 eligible patients, 379 (48.8%) were enrolled; 158 (41.7%) screened positive for FI. During the pandemic, there was a 18.1% relative increase (or 6.4% absolute increase) in the prevalence of FI in this population (P=0.040; OR=1.309, 95% CI 1.012–1.693). The majority (52.9%) of food-insecure subjects reported reduced access to food due to the pandemic. The most common perceived barriers to access to food were reduced food availability at grocery stores (31%), social distancing guidelines (26.5%), and reduced income (19.6%).

**Conclusion:**

Our findings suggest that nearly half of the clinically stable patients who presented to our urban ED during the pandemic experienced food insecurity. The prevalence of FI in our hospital’s ED patient population increased by 6.4% during the pandemic. Emergency physicians should be aware of rising FI in their patient population so that they may better support patients who must choose between purchasing food and purchasing prescribed medications.

## INTRODUCTION

The Life Sciences Research Office defines food insecurity (FI) as existing “whenever the availability of nutritionally adequate and safe foods or the ability to acquire acceptable foods in socially acceptable ways is limited or uncertain.”^1^ In 2016, 41 million Americans lived in food-insecure households.^2^ Adults experiencing FI have greater rates of office visit use, inpatient hospital stays, and emergency department (ED) visits.^3^,^4^ Reducing FI may lower healthcare service utilization and spending during major health crises.^4^

Food insecurity has also been shown to increase the risk of chronic disease, placing individuals at enhanced risk for complications due to COVID-19 infection.^5^ A recent study conducted by McDonough et al before the COVID-19 outbreak discovered that 35.3% of our urban teaching hospital’s ED patient population at MedStar Washington Hospital Center (MWHC) experienced FI.^6^ Screening for FI during the pandemic is an important first step in identifying the populations that are at highest risk of experiencing worse health outcomes. This data can be further used to direct healthcare spending during a crisis.

Globally, COVID-19 made access to staple foods and availability of fresh produce more challenging.^7^ Financially insecure families relied on complicated food purchasing methods and FI coping strategies. For example, many destitute households needed to travel long distances to acquire affordable food products. These families depended heavily on public transport and rideshare programs, both of which became risky modes of transportation during the pandemic. Additionally, during the outbreak, social distancing guidelines made sharing meals with family difficult, as well as made group meals at senior homes and soup kitchens nearly impossible.^8^ Furthermore, at the start of the pandemic, there was an upsurge in panic-buying, during which many families stockpiled food and supplies. This led to market shortages and rising prices.^9^ An increased incidence of food hoarding negatively affected low-income individuals’ access to food since these individuals lacked the financial means to buy products in bulk.^8^

The prevalence of FI and hunger in the ED population has historically been higher than among the general public.^2^,^10^ Therefore, the ED environment represents a unique opportunity for physicians to identify patients with FI. Our goal in this study was to assess the impact of the COVID-19 pandemic on the prevalence of FI in the ED patient population. We hypothesized that the prevalence of FI in the ED patient population significantly increased during the pandemic.

## METHODS

### Study Design

We conducted a single-center, observational, survey-based study in accordance with the Strengthening the Reporting of Observational Studies in Epidemiology (STROBE) Guidelines.^11^ The study protocol was reviewed and approved by the MedStar Health Research Institute Institutional Review Board. Subjects were enrolled from October 26–December 2, 2020 between the hours of 8 am–8 pm Monday through Friday (excluding November 26, 27, and 30 due to the Thanksgiving holiday) at an urban, adult, tertiary care teaching hospital ED with approximately 90,000 annual visits. In the pre-pandemic study, subjects were enrolled from November to December 2019 between the hours of 8 am–8 pm Monday through Friday in the same hospital ED.

**Population Health Research Capsule**

What do we already know about this issue?

*Food insecurity (FI) is associated with poor health outcomes. Many families had reduced access to food during the COVID-19 pandemic*.

What was the research question?

*Did the prevalence of FI among emergency department (ED) patients increase during the COVID-19 pandemic?*

What was the major finding of the study?

*The prevalence of FI in ED patients increased by 6.4% (absolute) and 18.1% (relative) during the COVID-19 pandemic (P=0.040, 95% CI 1.012–1.693)*.

How does this improve population health?

*By raising awareness of rising FI during the pandemic, our research may support increased funding for food banks and food pharmacies during global health crises*.

All clinically stable adult ED patients were approached by trained research assistants (RA). Verbal consent was chosen to minimize direct contact and exchange of materials between participants and study personnel during the pandemic and to reduce participation bias. Consenting participants were provided with an information sheet detailing their involvement in the study. Non-English speakers, patients presenting with altered mental status, clinically unstable patients, prisoners or patients in police custody, and patients <18 years were excluded from the study.

### Procedures for Data Collection

Research assistants used the electronic health records (EHR) to identify patients who met all inclusion and exclusion criteria and approached participants after they were seen by the treating clinician and prior to their disposition. After describing the study, RAs verbally administered a survey at the bedside of consenting participants.

The first two survey questions were taken from the previously validated Hunger Vital Sign screening tool for FI: 1) “Within the past 12 months, we worried whether our food would run out before we got money to buy more”; and 2) “Within the past 12 months, the food we bought just didn’t last and we didn’t have money to get more.”^12^ A response from the patient of “often true” or “sometimes true” to either question was categorized as a positive screen for FI, and the subject was subsequently provided a handout of community resources.^12^ To ascertain whether FI was influenced directly by the pandemic, we created the following additional questions: 3) “Has the COVID-19 pandemic changed your access to food?” and 4) “Which of the following factors reduced your access to food during the COVID-19 pandemic?” Lastly, we asked for the following socioeconomic variables: living situation; highest education level; employment; and household annual income. The only difference between the study designs for the pre-pandemic and intra-pandemic studies was that survey questions #3 and #4 were not included in the pre-pandemic study survey.

Using the EHR, we collected the following baseline characteristics: age; gender; race/ethnicity; weight; height, body mass index; pre-existing comorbid conditions; medication use for comorbid conditions; and history of substance use. Vital signs at presentation were used to screen for clinical stability. A de-identified log with no protected health information was kept to account for every patient who presented to the ED during the study period. We collected and managed study data on a secure tablet using REDCap electronic data capture tools hosted at MedStar Health.

Of note, our study design was nearly identical to that of McDonough et al.^6^ The only difference in our data collection methods was that the McDonough study took place in late 2019 (before the pandemic), while our study took place exactly one year later in late 2020 (during the pandemic).

### Data Analysis

The primary outcome of our study was the prevalence of FI in the ED patient population during the COVID-19 pandemic. Secondary outcomes included 1) the percentage of ED patients reporting reduced access to food due to the COVID-19 pandemic, and 2) patient-perceived barriers to accessing food during the COVID-19 pandemic. We evaluated patient characteristics with descriptive statistics and frequency distributions. Categorical traits were compared using the chi-square test or Fisher’s exact test. We compared continuous traits using the independent samples *t*-test or Wilcoxon rank-sum test. Statistical analysis was performed with SPSS 26 (IBM Corp, Armonk, NY).

### Vocabulary

In the remainder of this paper, McDonough et al’s 2019 study is referred to as the pre-pandemic study, while our 2020 study is referred to as the intra-pandemic study.

## RESULTS

### Enrollment and Patient Characteristics in the Intra-pandemic Study

**Figure 1:**
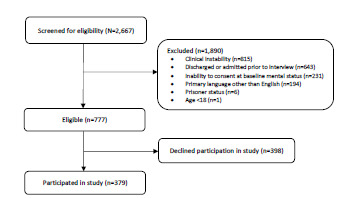
Participant enrollment in intra-pandemic study.

In total, 2,667 patients were screened for the study. Of those, 1,890 did not meet study criteria. Of the 777 eligible visits, 398 patients declined participation, resulting in a cohort of 379 (48.7%) participants (Figure 1). The characteristics of the study participants are presented in Table 1.

### Survey Answers in the Intra-pandemic Study

Of 379 subjects, 158 (41.7%) reported experiencing FI in the prior year. Table 2 summarizes the participants’ survey answers in the intra-pandemic study; 35% of all participants reported that the COVID-19 pandemic had reduced their access to food. Participants reported that the following factors reduced their access to food during the pandemic: reduced food availability at grocery stores (31.0%); social distancing guidelines (26.5%); reduced income (19.6%); reduced access to transportation (18.3%); unemployment (17.2%); illness or additional healthcare costs (9.8%); and other factors (3.7%) (Table 2). Examples of other self-reported factors included increased food hoarding, rising food prices, and reduced reliability of food delivery services. Additionally, among subjects experiencing FI, 26.7% reported that they had to choose between buying food and buying prescription medication over the prior 12 months (Table 2).

### Comparing the Pre-pandemic and Intra-pandemic Studies

The pre-pandemic study enrolled 685 total participants, 242 (35.3%) of whom were experiencing FI. The intra-pandemic study enrolled 379 total participants, 158 (41.7%) of whom were experiencing FI. This indicates a 6.4% absolute increase (18.1% relative increase) in the prevalence of FI (chi-square analysis, *P*=0.040, odds ratio [OR] 1.309, 95% CI 1.012–1.693).

### Factors Associated with Food Insecurity in the Intra-pandemic Study

We used contingency coefficients (C) to measure the magnitude of association between specific patient characteristics and FI. During the pandemic, FI in our ED patient population was moderately associated with race (C=0.209), employment status (C=0.236), annual income level (C=0.348), and household situation (C=0.298); weakly associated with substance use history (C=0.182); and not associated with gender (C=0.036).

### Logistic Regression Results

We performed a binary logistic regression to identify which patient variables may have influenced the effect of the pandemic on FI. The results of the logistic regression are detailed in Table 3. All the listed predictor variables are included in the model.

## DISCUSSION

### Rise in Food Insecurity

**Figure 2:**
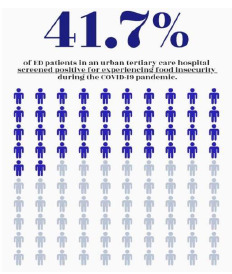
Prevalence of food insecurity in the emergency department patient population during the COVID-19 pandemic.

Our study found that the prevalence of FI in the ED patient population during the COVID-19 pandemic was 41.7% (Figure 2). This is more than quadruple the national prevalence of FI, as well as nearly triple the prevalence of FI in Washington, DC.^13^,^14^ This suggests that those who use our ED during health crises are more likely experiencing FI when compared to the general population. During the COVID-19 pandemic, there was an absolute 6.4% increase (or 18.1% relative increase) in the prevalence of FI in our ED patient population.^6^ The majority of patients experiencing FI reported that the pandemic had reduced their access to food, whereas the majority of those not experiencing FI reported that the pandemic had not changed their access to food. This supports that the pandemic played a role in worsening FI among ED patients.

The most common perceived barriers to accessing food during the pandemic (in descending order) included reduced food availability at grocery stores, social distancing guidelines, reduced income, reduced access to transportation, and unemployment. This reveals that the socioeconomic strains imposed during a pandemic may worsen FI among patients seeking acute care. To reduce FI during future health crises, it would be worthwhile to support guidelines that limit panic-buying and promote safe transportation practices.

It is important to note, however, that the association between the pandemic and FI was only as strong as the pandemic’s relationship with other patient variables. As seen in the logistic regression, there was a shared association between the pandemic and other patient traits (Table 3).

### Demographics Associated with Experiencing Food Insecurity

Patients who screened positive for FI were significantly more likely to rent or live with family rather than own their own home, obtain a high school degree or General Education Diploma rather than pursue post-secondary education, be unemployed or work part-time rather than work full-time, and have a household annual income <$50,000 (Table 1). This suggests that ED patients experiencing FI have lower rates of home ownership, higher education, full-time job opportunities, and overall income. Individuals experiencing FI face competing needs for food, shelter, and education.

### Changes During the Pandemic

In comparison to the pre-pandemic study, our intra-pandemic study had significantly higher proportions of participants who lived with family rather than independently. This suggests that the financial restraints of the COVID-19 pandemic may have limited an individual’s ability to afford living on their own. Our intra-pandemic study also had significantly higher proportions of participants who obtained higher education (36.2% pre-pandemic vs. 63.8% intra-pandemic, *P*<0.01), worked full-time (21% pre-pandemic vs. 47.3% intra-pandemic, *P*<0.01), and had a household annual income >$50,000 (8.4% pre-pandemic vs. 41.7% intra-pandemic, p<0.01). This indicates that individuals of lower socioeconomic status were less likely to present to the ED during the pandemic. It is plausible that this patient population faced more challenges in traveling to the ED for medical care during the health crisis. Examples of such challenges may have included limited transportation options, inability to take time off work, or reduced income to pay for medical bills. This population may also have been more hesitant to visit the ED during the pandemic out of fear of contact with sick individuals. It is also possible that they were less likely to consent to participate.

### Implications

Food insecurity has become a strong predictor for a decline in overall health including in the development of chronic conditions, many of which are preventable.^15^ In addition to a decline in physical health, the development of mental health disorders (such as anxiety and depression) have also been linked to FI.^16^ Moreover, nutrient deficiencies can weaken immune defense mechanisms, increasing susceptibility to infections^17^ such as COVID-19, making the relationship of causality difficult to discern.

Food insecurity and COVID-19 have demonstrated a bidirectional relationship where one exacerbates the other, disproportionately affecting vulnerable populations.^18^ The COVID-19 pandemic negatively impacted the economy, resulting in record unemployment and underemployment rates. As a result, FI rates increased in the general population, most notably among those who work in lower wage positions that are most vulnerable to job losses.^19^ Individuals already living in poverty may live in environments, such as crowded multigenerational housing, that increase the risk for COVID-19 exposure. The effects of COVID-19 have had compounded effects upon the livelihood of vulnerable populations. It is imperative for health systems to recognize this relationship and to provide aid at the appropriate level.

On a larger level, FI is associated with exacerbations of chronic diseases and increased health expenditures.^4^ As more individuals become infected with COVID-19, more patients will use the healthcare system. Food insecurity has been shown to be linked to an increased number of ED visits, hospitalizations, and extended hospital stays.^5^ However, the full socioeconomic impact of COVID-19 may not immediately be evident as the effects of FI on one’s overall health take time to develop.

## LIMITATIONS

A number of limitations arose during the completion of this observational study. First, inclusion and exclusion criteria for participant selection challenged the generalizability of our study results. For example, non-English speaking patients, pediatric patients, prisoners, and patients in police custody were not included (Figure 1). In turn, results may not be applicable to a more diverse patient population. Similarly, patients were required to be clinically stable without alteration in mental status to participate, which may have introduced bias toward healthier patients. With data collection limited to a short time frame and occurring only during the daytime on weekdays, patients presenting overnight, on weekends, and during other times of the year were not included in the study, also potentially hindering the generalizability of results. Of note, however, the same inclusion and exclusion criteria and collection times were implemented in the pre-pandemic and intra-pandemic studies, limiting some of these sources of bias and suggesting the populations were comparable.

Additionally, our study’s sample size was 44.7% smaller than in the McDonough study. This significant reduction in sample size lowered the power of our study. Notably, our small sample size likely contributed to the small effect size that described the pandemic’s influence on FI. Due to fears of contracting COVID-19, many families avoided visiting the ED in 2020.^20^ As a result, the overall reduction in ED volume in late 2020 probably led to fewer eligible study participants in our study, contributing to our significantly reduced sample size.

Logistics of conducting a clinical study in the time of the COVID-19 pandemic introduced further limitations to the study. As mentioned above, surveys were conducted electronically by RAs to minimize physical exchange of material. And as with any study using patient-reported responses, there is potential that results were skewed by recall bias. However, an additional risk of response bias was also introduced as patient responses were collected verbally. With stigma surrounding FI, the need to verbally report answers regarding patient experience with FI to RAs (as opposed to independent completion of paper surveys) may have led to under-reporting and falsely low prevalence of FI among our participants. Additionally, many patients chose not to participate, limiting the sample size considerably.

Many of the RAs were discouraged from or elected not to enter rooms designated for patients who tested positive for COVID-19. This further limited our sample size and led to inclusion of a healthier participant group. To limit the exposure of RAs to potential COVID-19 infection while in the ED, only one RA was present in the ED at a time. With only one RA available for data collection, some individuals presenting to the ED were admitted to the hospital or discharged before they could be approached for study participation. Additionally, unlike in past years, RAs spent additional time during the pandemic appropriately donning and doffing personal protective equipment for each patient interaction. This task likely limited the amount of time they had to visit each ED patient before they were discharged or admitted.

## CONCLUSION

The prevalence of ED patients experiencing food insecurity at a high volume, urban, tertiary care center increased by 18.1% (6.4% absolute) during the pandemic. Most participants experiencing FI reported that the pandemic had reduced their access to food. Common perceived barriers to accessing food during the pandemic included reduced food availability at grocery stores, social distancing guidelines, and reduced income. It is important to make emergency physicians aware of the rising prevalence of FI so that they may better support the increased number of ED patients who must choose between purchasing food and purchasing prescribed medications. This research provides insight into the rising prevalence of FI and increasing barriers to access of food during the challenging COVID-19 pandemic. We hope our findings will provide evidence in support of increased funding for nationwide food banks and food pharmacies. Looking ahead, our findings may be used as pilot data for a larger study and help inform health policy. This work enhances our knowledge of the current health crisis’ influence on health disparities, as well as on social determinants of health in the practice of emergency medicine.

## Supplementary Information



## Figures and Tables

**Table 1:** Patient characteristics in intra-pandemic study.

Characteristic	Food-insecure n=158 (41.7%)	Non-food insecure n=221 (58.3%)	P-value
Average age	48.5	51.8	>0.05
% Female	57.7%	51.1%	0.139
Race			<0.01
Black	135	161	
White	5	41	
Hispanic/Latinx	10	9	
Other	6	12	
Household situation			<0.01
Own	12	60	
Rent	89	96	
Live with family	46	56	
Senior home	1	3	
Senior nursing facility	0	1	
Undomiciled	7	2	
Declined to state	3	2	
Highest education level			<0.01
High School/GED	92	75	
Some college	31	56	
Associates	6	11	
Highest education level			
Bachelors	6	36	
Masters/Doctorate	5	38	
Trade/Apprenticeship	8	5	
Declined to state	7	0	
Employment			<0.01
Full-time	33	104	
Part-time	16	16	
Unemployed	65	36	
Disabled	23	18	
Retired	20	44	
Declined to state	0	2	
Household annual income			<0.01
<$12,490	43	23	
$12,490–$24,999	26	26	
$25,000–$49,999	29	30	
$50,000–$74,999	12	32	
$75,000–$99,999	0	24	
>$100,000	1	37	
Declined to state	43	51	
History of substance use	27%	17.3%	0.02
Average SBP	137.5	136.2	0.172
Average glucose level	131.8	121.7	0.756
Average BMI	31	30.2	0.329
Past medical history of:			
Hypertension	40.4%	40.8%	0.934
Hyperlipidemia	19.9%	20.2%	0.941
Diabetes mellitus	26.9%	19.7%	0.1
Coronary artery disease	11.5%	9%	0.412
Cancer	3.2%	5.8%	0.237
Obesity	43.6%	40.8%	0.589
On medications for:			
Hypertension	34.6%	32.7%	0.703
Hyperlipidemia	16.7%	17.5%	0.834
Diabetes mellitus	19.9%	14.8%	0.194
Coronary artery disease	16%	9.4%	0.053

*GED*, General Education Diploma; *BMI*, body mass index; *SBP*, systolic blood pressure.

**Table 2:** Survey answers describing the influence of the COVID-19 pandemic on participants’ access to food.

Characteristic	Food-insecure n=158 (41.7%)	Non-food insecure n=221 (58.3%)	P-value
Has the COVID-19 pandemic changed your access to food?			
Yes. I have less access to food.	82 (52.9%)	50 (22.4%)	
No. There has been no change in my access to food.	57 (36.8%)	155 (69.5%)	
Yes. I have more access to food.	16 (10.3%)	18 (8.1%)	<0.01
Which of the following factors reduced your access to food during the COVID-19 pandemic?			
Reduced food availability at grocery stores	117 (31%)	N/A	
Social distancing guidelines	100 (26.5%)	N/A	
Reduced income	74 (19.6%)	N/A	
Reduced access to transportation	69 (18.3%)	N/A	
Unemployment	65 (17.2%)	N/A	
Illness or additional healthcare costs	37 (9.8%)	N/A	
Other	14 (3.7%)	N/A	
In the last 12 months, have you ever had to choose between buying food and buying prescription medication?*			
Yes	33 (26.7%)	8 (3.7%)	<0.01

* A total of 335 subjects (124 food-insecure subjects and 219 non-food insecure subjects) chose to respond to this survey question. In other words, 44 of the total study participants elected not to answer this question. As a result, the percentages in this row are adjusted to reflect 335 total subjects (not 379 total subjects). *COVID-19*, coronavirus disease 2019.

**Table 3:** Logistic regression.

Variable(s)	P-value	Odds Ratio	95% Confidence Interval
Pandemic	0.777	1.052	(0.740–1.495)
Gender	0.275	1.207	(0.861–1.693)
Race	0.001	3.029	(1.589–5.775)
Employment	0.005	1.808	(1.200–2.724)
Income	<0.0001	2.472	(1.685–3.628)
Household situation	<0.001	4.363	(2.705–7.037)
Substance use history	0.001	2.077	(1.329–3.245)
